# Internalization of *Leptospira interrogans* via diverse endocytosis mechanisms in human macrophages and vascular endothelial cells

**DOI:** 10.1371/journal.pntd.0010778

**Published:** 2022-09-22

**Authors:** Xin Zhao, Jun Guo, Xiaoyuan Jia, Yaling Yang, Lijuan Liu, Weizhong Nie, Zhiqiang Fang

**Affiliations:** 1 Institute of Health Quarantine, Chinese Academy of Inspection and Quarantine, Beijing, China; 2 State Key Laboratory of Food Nutrition and Safety, Key Laboratory of Food Quality and Health of Tianjin, Tianjin University of Science & Technology, Tianjin, China; 3 College of Life Sciences and Medicine, Zhejiang Sci-Tech University, Hangzhou, China; 4 Department of health quarantine, Qinhuangdao Customs District, Qinhuangdao, China; UConn Health, UNITED STATES

## Abstract

Leptospirosis, one of the leading global causes of morbidity and mortality, is an emerging public health problem, particularly in large urban centers of developing countries. Leptospirosis results from infection with an organism belonging to the *Leptospira* genus *L*. *interrogans*. The extensive invasive ability has previously been documented, however a mechanism that describes how the organism is internalized by human macrophages and transmigrates through human blood vessel remains poorly understood. In the present study, we utilized a human macrophage and vascular endothelial cell line to study the diverse invasive mechanisms by which *L*. *interrogans* infections occur. We found that THP-1 and HUVEC had a diverse expression of cell receptors and *L*. *interrogans* entered THP-1 and HUVEC by different pathways. In the macrophage model cell line, ITGB1/FAK-signaling mediated microfilament dependent endocytosis with lysosome fusion, whereas ITGB1/CAV-1/PI3K-signaling mediated microfilament dependent endocytosis and transcytosis without lysosome fusion in the endothelial cell model. Shedding of pathogenic leptospires from HUVEC displayed higher viability than those from THP-1. The monolayer of HUVEC maintained integrity during the infection, while 3D imaging showed that leptospires were transmigrated both intra- and intercellularly. These results indicate that endocytosis of leptospires in human macrophages and human vascular endothelial cells are quite different, macrophages are responsible for eliminating leptospires in the human body during the infection while vascular endothelial cells facilitate dissemination of leptospires from blood vessels into target organs where they cause injury.

## Author summary

Leptospirosis is a zoonotic bacterial disease which causes 1.03 million cases and 58,900 deaths each year. Human infections occur when the primary reservoir hosts, such as rodents, contaminate food and water with leptospires. Unlike other bacterial pathogens, leptospires invade the human body through mucosal barriers and enter the bloodstream, which can result in septicemia. Left untreated, leptospirosis can spread into multiple organs and tissues such as lungs, liver and kidneys. Pathological features of this disease include high fever, myalgia, lymphadenectasis, hemorrhaging and jaundice. Human macrophages and vascular endothelial cells play important roles in eliminating and preventing the transmission of this pathogen. We speculated that leptospires could be neutralized in macrophages and subsequently transported by vascular endothelial cells throughout the human body. In this research, the diverse mechanisms of human macrophages and vascular endothelial cells infected by leptospires were explored. Our findings can be used to improve the treatment, prevention, and supervision regarding leptospire transmission and infection.

## Introduction

Leptospirosis, caused by infection with pathogenic *Leptospira* species, is a zoonotic disease of global importance endemic to Southeast Asian and South America. An estimated 1.0 million cases of leptospirosis and 58,900 deaths occur worldwide annually, most taking place in low income urban and rural areas that lack the capacity to cope with floods, prevent the stagnation of water, and perform proper rodent control [1–5]. However, leptospirosis cases have recently been reported in Europe and North America resulting in *Leptospira* being considered as an emerging or re-emerging disease in these areas [6,7].

Many animals, such as rodents, dogs and livestock, serve as the natural hosts of pathogenic *Leptospira* species [8,9]. Transmission of the spirochete from animals to humans occurs indirectly through contact with soil or water that had been contaminated with the leptospire-contaminated urine of infected animals [10,11]. After invasion into the human body through the skin and mucosa, the leptospires enter the blood stream rapidly to cause septicemia, and then spread into internal organs such as lungs, liver and kidneys within about a week after infection [12,13]. The subsequent clinical manifestations can include septic shock, pulmonary diffuse hemorrhage and renal failure depending on the primary organs impaired [14,15]. However, the molecular and cellular basis in pathogenicity of the spirochete remains poorly understood.

Infection is a process of the interaction between microbial pathogens and hosts, and the interactions of which will decide the survival of both host cell and pathogen [16,17]. Mononuclear macrophages have been shown to phagocytize leptospires, and can kill the phagocytosed intracellular leptospires, a process by which *L*. *interrogans* evades by promotion of apoptosis and necroptosis in monocytes [18,19]. Like many bacterial pathogens capable of causing systemic infection, pathogenic leptospires can pass through blood vessel walls without disrupting the integrity of vessel and disseminate from the blood stream into the target organs where they cause tissue injury [20].

Adherence to a target host cell is the initial step of infection for microbial pathogens. Previous reports showed that pathogenic leptospires could adhere to several extracellular matrix (ECM) molecules such as fibronectin (FN), laminin (LN), collagen I (COL1) and COL4 [21–23]. Allosteric remodeling of the host ECM molecules occurs after interaction with microbial adhesins, which enable these molecules to bind to the N-terminal of integrins in the cell membrane. Integrins, composed of two subunits (α and β), can be classified into three subfamilies (ITGB1, ITGB2 and ITGB3) based on the difference of β-subunits [24]. Outer membrane proteins of leptospires, including LigA, LigB, Lsa21, Lsa24 and Loa22 are adhesins that carry out the interaction with ECM of the host organism [25–27]. The combination of integrins with their ligands causes the formation of integrin dimerides to trigger either FAK- or PI3K-based intracellular signaling which results in the internalization of microbial pathogens [28,29]. Alternatively, the polymerization of integrins can also activate caveolin-1, a major component of caveolae in vascular endothelial cells, myocytes and pneumocytes. Activation of caveolin-1 mediates the internalization of bacterial pathogens into host cells by formation of caveolae-based phagocytotic vesicles. The process of caveolae internalization regulates integrin-dependent signaling pathways [24,30–32]. Previous studies revealed that pathogenic *Leptospira* species enter host cells by endocytosis [33]. The mammalian cell entry (Mce) protein of pathogenic Leptospira is an RGD (Arg-Gly-Asp)-motif-dependent virulence factor capable of interacting with host ECM components and can directly bind to integrins such as α5β1, αvβ3, αMβ2 and αLβ2. The interaction between Mce and these integrins can activate intracellular signaling of integrin, leading to cytoskeletal rearrangements that result in microbial internalization [34,35]. Until now however, the role of caveolins in leptospiral internalization into host cells has not been reported yet.

Among serogroups of pathogenic *Leptospira* species, *L*. *interrogans* serogroup Icterohaemorrhagiae has been shown as the most prevalent in the world [36]. In China, about 70% of leptospirosis patients are infected with the *L*. *Icterohaemorrhagiae* serogroup [37]. In the present study, we detected integrins and caveolins expressed by a human monocyte line (THP-1) and an umbilical vein endothelial cell line (HUVEC). The roles of different intergrins and caveolins as well as intergrin- or caveolin-triggered signaling pathways in internalization of the spirochete were subsequently determined. The results of this study demonstrated the various mechanisms different host cells employ to internalize pathogenic *Leptospira* species. We found human macrophages and human vascular endothelial cells express a diversity of integrins and caveolin which trigger different pathways, ITGB1/FAK-signaling mediated microfilament dependent endocytosis fused with lysosomes in macrophages, while ITGB1/CAV-1/PI3K-signaling mediated microfilament dependent endocytosis and transcytosis without lysosome fusion in vascular endothelial cells. Leptospires from the human vascular endothelial cells showed higher viability than those from the macrophages. We speculate the lossless internalization and transcytosis of leptospires in vascular endothelial cells facilitates dissemination of leptospires from blood vessels into target organs where they cause injury in human body.

## Materials and methods

### Leptospiral strain and culture

*L*. *interrogans* serogroup Icterohaemorrhagiae serovar Lai strain Lai which is a high-passage strain was provided by the National Institute for Control of Pharmaceutical and Biological Products in Beijing, China. The strain was cultivated at 28°C in Ellinghausen-McCullough-Johnson-Harris (EMJH) liquid medium supplemented with 5% albumin bovine fraction V (Sigma, USA) and 0.05% Tween-80 (Difco, USA).

### Cell lines and culture

A human monocytic cell line (THP-1) and a human umbilical vein endothelial cell line (HUVEC) were provided by the Cell Bank of the Institute of Cytobiology, Chinese Academy of Science, Shanghai, China. The cells were maintained in RPMI 1640 liquid medium (Gibco, USA), supplemented with 10% fetal calf serum (FCS, Gibco), 100 U ml^-1^ penicillin (Sigma, USA) and 100 μg ml^-1^ streptomycin (Sigma, USA) at 37°C in an atmosphere of 5% CO_2_. In particular, THP-1 cells were pre-treated with 10 ng ml^-1^ PMA (Sigma) at 37°C for 48 h to differentiate them into macrophages before use.

### Detection of intracellular leptospires by transmission electron microscopy

Cells were infected with *L*. *interrogans* strain Lai with a multiplicity of infection (MOI) of 100 (100 leptospires per host cell) at 37°C for 1 h. After washing with PBS and fixation with 2.5% glutaraldehyde-PBS for 2 h, the cells were scraped from the wells for a 10-min centrifugation at 250×g. The cell pellets were postfixed, dehydrated, embedded in TAAB resin, ultrathin sectioned and stained. The intracellular leptospires were observed under a transmission electron microscope (type TECNAI-10, Philips, Holland).

### Detection of intracellular leptospires by confocal microscopy

Cells were infected with *L*. *interrogans* strain Lai with a multiplicity of infection (MOI) of 100 at 37°C for 0.5, 1, 2 or 4 h. After treatment with 50 μg ml^-1^ gentamycin for 15 min to kill the extracellular leptospires and with 0.25% trypsin-PBS for 5 min to detach the cell-adhered leptospires, the cells were centrifuged at 100×g for 10 min at 4°C. The cell pellets were fixed with 4% glutaraldehyde-PBS for 30 min, and then permeabilized with 0.1% TritonX-100-PBS for 10 min to allow antibody penetration into the cells. After washing with PBS and blockage with 5% BSA-PBS, the cells were incubated with rabbit anti-*L*. *interrogans* strain Lai-IgG, followed by incubation with Alexa Fluor594**-**conjugated donkey anti-rabbit-IgG (Invitrogen) for 1 h to stain intracellular leptospires. After washing with PBS again, the cells were incubated with 1 μg ml^-1^ DAPI (Sigma) for 10 min to stain cell nucleus. Finally, the cells were smeared on glass slides and then observed under a laser confocal microscope (type FV1000, Olympus).

### Detection of cytoskeletal rearrangement in cells during infection

THP-1 cell or HUVEC (1×10^5^ per well) were seeded in 12-well plates (Corning) for a 24-h incubation at 37°C. After washing with PBS, the cells were infected with *L*. *interrogans* strain Lai at a MOI of 100 for 1 or 2 h. Confocal microscopy were used to detect microfilament and microtubule with phalloidin-FITC (Sigma) and rat anti-tubulin-IgG (Abcam) as method above. Finally, the cells were smeared on glass slides and then observed under a laser confocal microscope (type FV1000, Olympus). Spot number of polymerized actin and tubulin were mearsured by Feret’s diameter using Image J software (version 1.53e).

### Determination of viability of intracellular leptospires

THP-1 or HUVEC (1×10^6^ per well) were seeded in 6-well culture plates (Corning) for a 24-h incubation at 37°C. After washing with PBS, the cells were infected with *L*. *interrogans* strain Lai at a MOI of 100 (1×10^8^) for 1, 2 or 4 h. The cells were treated with gentamicin and trypsin as above. After repeated washing with PBS and centrifugation at 400×g for 10 min (4°C), the cell pellets were harvested for lysis with cold autoclaved 0.05% SDC-PBS. The lysates were centrifuged at 400×g for 10 min (4°C) to remove cell debris. The supernatants were collected to centrifuge at 17,200×g for 15 min (4°C) to precipitate leptospires. The leptospiral pellets were suspended in EMJH liquid medium for counting leptospires with a Petroff-Hausser counting chamber (Fisher Scientific, USA) under a dark field microscope [38]. 100 μl of each of the suspensions containing 1×10^6^ leptospires was inoculated into 900 μl EMJH medium for a 6-d incubation at 28°C for counting as previously described [39]. In this assay, the same number of leptospires from EMJH medium was used as the control.

### Detection of cellular integrins and CAV-1 by flow cytometry

Integrin β1, β2 or β3 subunit and CAV-1 expressed by host cells were detected. THP-1 or HUVEC (1×10^6^) were fixed and permeabilized with a commercial fixation and permeabilization agent (eBioscience, USA), and then blocked with 2% donkey serum for 15 min at 4°C. Using goat anti-integrin β1, β2 or β3 subunit-IgG (Santa Cruz) and rabbit anti-CAV-1-IgG (Abcam) as the primary antibody, isotype protein is used as control antibody. Alexa Fluor488-conjugated donkey anti-goat-IgG (Invitrogen) and Alexa Fluor488-conjugated donkey anti-rabbit-IgG (Invitrogen) as the secondary antibody, the ITGB1, ITGB2, ITGB3 or CAV-1 expressed by the cells were detected by flow cytometery (type FC500MCL, Beckman, Germany).

### Generation and identification of target gene-depleted cell lines

Integrin subunit β1-, β2- or β3-encoding gene of THP-1 cells or HUVEC (1×10^5^), and CAV-1-encoding gene of HUVEC (1×10^5^) were depleted using a siRNA Transfection Kit (Thermo Scientific) with 5–30 μM Stealth select siRNAs from [Home sapiens] according to the manufacturer’s protocols. Real-time fluorescence quantitative RT-PCRs were performed to determine the depletion of the target genes using a RT reagent Kit (TaKaRa) for transcription and a SYBRPremix Ex-Taq II Kit (TaKaRa) for amplification, in which the GAPDH-encoding gene was used as the control. The primers used in the RT-PCRs were listed in Table 1.

**Table 1:** Primers used in the real-time quantitative PCRs.

Gene	Sequence (5’ to 3’)
human ITGB1	F: GGAGAGTGCGTCTGCGGA
	R: TGCCAGTGTAGTTGGGGTTG
human ITGB2	F: TTGGCTTCGGGTCCTTCG
	R: TGGCACTCTTTCTCCTTGTTGG
human ITGB3	F: GGAGGTTTGTTTAGAAGAAGTGTGT
	R: TGTGGAGGTGCTATGGATGAG
human CAV-1	F: GCAGAACCAGAAGGGACACAC
	R: GCAGACAGCAAGCGGTAAAAC
human GAPDH	F: ACGGATTTGGTCGTATTGGG
	R: CGCTCCTGGAAGATGGTGAT

F: forward primer; R: reverse primer

### Determination of roles of integrins and CAV-1 in internalization of leptospires

Integrin subunit β1-, β2-, β3- or CAV-1 were depleted by siRNA or inhibited by antibody or inhibitor. The integrin subunit β1-, β2-, β3- or CAV-1-encoding gene depleted, 30 μg antibody against integrin β1, β2 or β3 subunit (Santa Cruz) or 1.5 nM of a caveolae inhibitor filipin (Sigma) treated THP-1 or HUVEC cells were infected with *L*. *interrogans* strain Lai at a MOI of 100 for 1 h at 37°C. The subsequent steps and observation of intracellular leptospires by confocal microscopy were the same as described above.

### Analysis of relative gene expression of integrins and CAV-1 in THP-1 and HUVEC

THP-1 or HUVEC (1×10^6^ per well) were seeded in 6-well culture plates (Corning) for a 24-h incubation at 37°C. The cells were infected with *L*. *interrogans* strain Lai at a MOI of 100 for 4h at 37°C, cells without infection were used as control.

Total RNA was extracted with RNAiso Plus (9108, TaKaRa, Dalian, China) according to the manufacturer’s instructions. The Real-time PCR (RT-qPCR) detections were carried out with TB Green Premix Ex Taq (Tli RNase H Plus) to detected ITGB1, ITGB2, ITGB3, CAV-1 and GAPDH expression in THP-1 and HUVEC respectively, primers used were showed in Table 1. Fold change of ITGB1, ITGB2, ITGB3 and CAV-1 genes was calculated by the 2–ΔΔCt method [40].

### Detection of FAK and PI3K phosphorylation

THP-1 or HUVEC (1×10^6^ per well) were seeded in 6-well culture plates (Corning) for a 24-h incubation at 37°C. After washing with PBS, the cells were infected with *L*. *interrogans* strain Lai at a MOI of 100 for 0.5 or 1 h at 37°C. Cells were treated with 50 μg ml^-1^ gentamycin for 15 min to kill the extracellular leptospires and with 0.25% trypsin-PBS for 5 min to detach the cell-adhered leptospires, the cells were centrifuged at 100×g for 10 min at 4°C and subdivided in two for immunofluorescense and immunoblotting. Cells used for immunoblotting were lysed with RIPA lysis buffer (Millipore). FAK (Cell Signaling Tech, 71433), PI3K(Cell Signaling Tech, 4263), and phosphorylation of FAK (Tyr397, Cell Signaling Tech, 8556) and PI3K (Ser249, Cell Signaling Tech, 13857) were detected by westernblot. The immunoblotting signals reflecting protein phosphorylation were quantified by densitometry (gray scale determination) using an Image Analyzer (Bio-Rad). In the assays, THP-1 or HUVEC without infection were used as the controls.

### Generation of three dimensional transcytosis image of leptospires

HUVEC or (1×10^6^ per well) were seeded in 12-well culture plates (Corning) for a 24-h incubation at 37°C. The cell monolayers were infected with *L*. *interrogans* strain Lai at a MOI of 100 for 0.5, 1, 2, 4, 8, 12 or 24 h. After fixation with paraformaldehyde, permeabilization with TritonX-100 and blockage with BSA-PBS as above, the cell monolayers were incubated with rabbit anti-*L*. *interrogans* strain Lai-IgG, followed by incubation with Alexa Fluor594-conjugated donkey anti-rabbit-IgG (Invitrogen) for 1 h to stain intracellular leptospires. After washing with PBS, the monolayers were incubated with 30 μM of Dio dye (Beyotime, China) for 15 min to stain cells, and then imaged on a confocal microscope (type FV1000, Olympus) (590 nm excitation and 617 nm emission wavelengths for Alexa Fluor594 detection, and 484 nm excitation and 501 nm emission wavelengths for Dio dye detection) using AutoQuant X3 software.

### Transwell assay

To assess the potential roles of integrins and CAV-1 in transmigration of *L*. *interrogans* through small blood vessel wall, transwell assay was performed as previously described [41,42].

### Detection of co-localization of leptospires with lysosomes or caveolaes

Confocal microscopy-based co-localization assay was applied to assess the fusion of intracellular leptospires with lysosomes and the localization of intracellular leptospires in caveolaes. After washing with PBS, the cells were infected with *L*. *interrogans* strain Lai at a MOI of 100 for 1, 2 or 4 h. Rabbit anti-*L*. *interrogans* strain Lai-IgG, goat anti-LAMP-1-IgG (Santa Cruz) which is a lysosomal-associated membrane protein and rabbit anti-CAV-1-IgG (Abcam) were used to detect leptospires, lysosomes and caveolaes respectively by confocal microscope (type FV1000, Olympus). In this assay, THP-1 and HUVEC without infection were used as the controls.

### Detection of integrins and CAV-1 in phagocytic vesicles

THP-1 or HUVEC (1×108 per flask) were seeded in 50-ml culture flasks (Corning) for a 24-h incubation at 37°C. The cell monolayers were infected with *L*. *interrogans* strain Lai at an MOI of 100 for 1 h at 37°C. After treatment with gentamycin and trypsin as above, the cells were precipitated by a 10-min centrifugation at 100×g (4°C), and the leptospire-containing phagocytic vesicles in the cells were separated as previously described [43]. Briefly, the leptospire-infected cells were broken on ice using a glass homogenizer and then centrifuged at 400×g for 10 min (4°C) to remove cell debris. The harvested supernatants were mixed with 50 U Benzonase nucleinase (Sigma) and 1:100 diluted protease inhibitor mixture (Sigma) for a 5-min incubation at 37°C.

The mixtures were added with 65% sucrose solution to make the final sucrose concentration to 39%, and then a 5-step gradient sucrose solution (65%, 55%, 39%, 32.5% and 10%) was added one by one. After centrifugation at 100,000×g for 1 h (4°C), the phagocytic vesicles migrated into the 55% to 65% sucrose fractions. The fractions were collected to mix with homogenization buffer to 11% of the final sucrose concentration, and then placed on 15% Ficoll (Sigma) in a tube for a 20-min centrifugation at 18,000×g (4°C). The pellets were suspended in 10 ml homogenization buffer for another centrifugation step as above to harvest the pellets of phagocytic vesicles. The pellets were lysed with RIPA lysis buffer (Millipore), followed by a 400×g centrifugation at 4°C for 10 min to remove membrane debris. The supernatants were collected to measure protein concentration using a BCA Protein Assay Kit (Thermo Scientific). After SDS-PAGE and electro-transferring onto PVDF membrane (Millipore), the integrins and CAV-1 in the protein samples were detected by Western blot assay, in which goat anti-ITGB1, ITGB2 or ITGB3-IgG (Santa Cruz) or rabbit anti-CAV-1-IgG (Abcam) was used as the primary antibody, and HRP-conjugated donkey anti-goat-IgG (Santa Cruz) or goat anti-rabbit-IgG (Cell Signaling Techology) was used as the secondary antibody. In the assay, the normal goat or rabbit IgG (Santa Cruz; Abcam) instead of goat anti-ITGB-IgGs or rabbit anti-CAV-1-IgG as the primary antibody was used as the controls.

### Statistical analysis

Data from a minimum of three experiments were averaged and presented as mean ± standard deviation (SD). One-way analysis of variance (ANOVA) followed by Dunnett’s multiple comparisons test were used to determine significant differences. Statistical significance was defined as *p* <0.05.

## Results

### Endocytosis as the mechanism for internalization of *Leptospira* in THP-1 and HUVEC via microfilaments rearrangement

**Fig 1:**
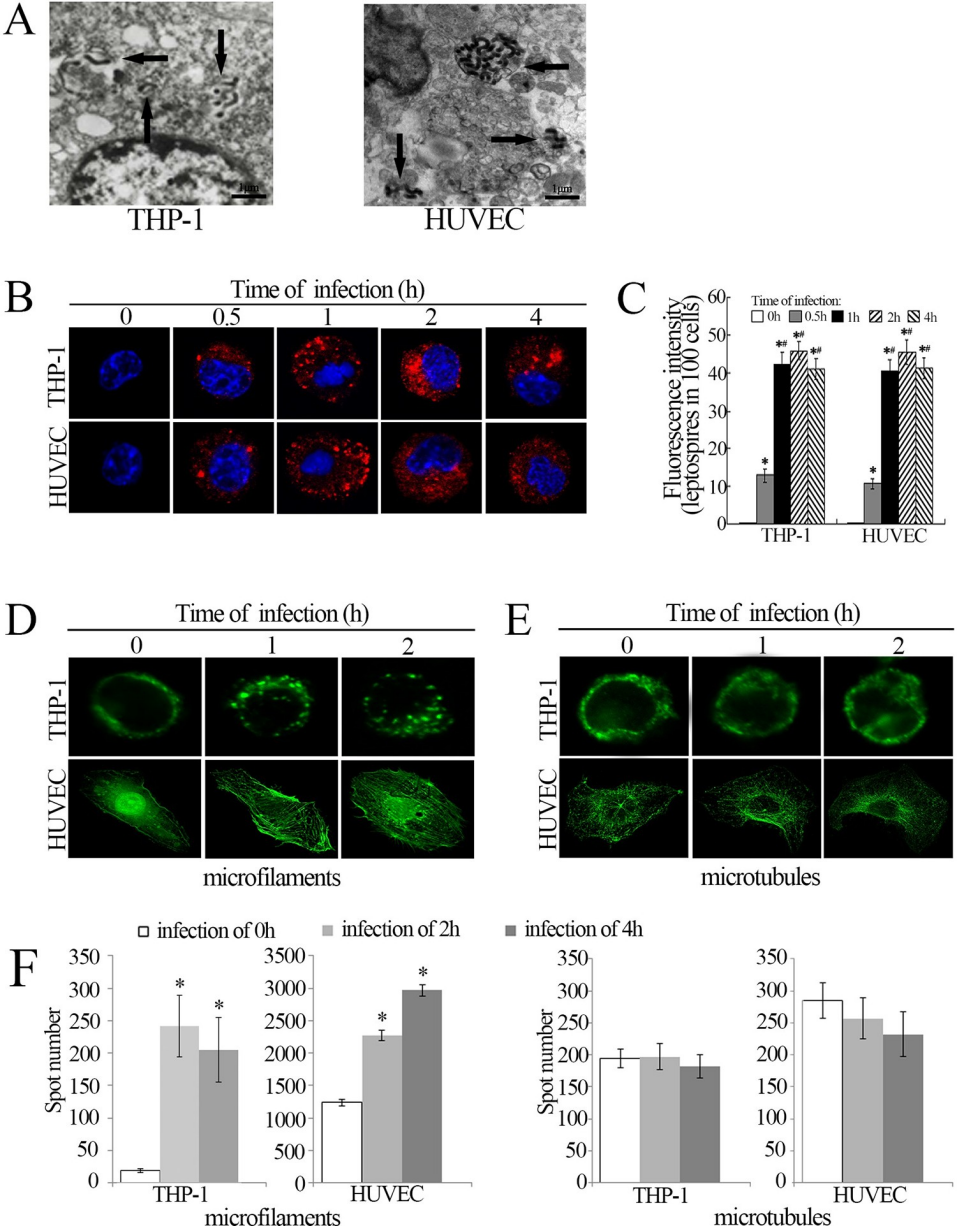
Internalization of *Leptospira* in THP-1 and HUVEC. A: Leptospires in THP-1 and HUVEC under the transmission electron microscope after infection with *L*. *interrogans* strain Lai for 1 h. The arrows indicate the intracellular leptospires; B: Leptospires in THP-1 and HUVEC under the laser confocal microscope after infection with *L*. *interrogans* strain Lai for the indicated times. The blue plaques in the middle of cells indicate the nuclus. The red spots around the nuclus indicate the intracellular leptospires; C: Statistical summary of red fluorescence intensity reflecting the leptospires in THP-1 and HUVEC after infection with *L*. *interrogans* strain Lai for the indicated times. Statistical data from experiments such as shown in B. Bars show the means ± SD of three independent experiments. The 0 h indicates the cells before infection. One hundred cells were analyzed for each of the specimens. *: *p* < 0.05 *vs* the red fluorescence intensity in the three cells before infection. ^#^: *p* < 0.05 *vs* the red fluorescence intensity reflecting the leptospires in the cells after infection with the spirochete for 0.5 h; D: Microfilament aggregation in THP-1 and HUVEC during infection with *L*. *interrogans* strain Lai for the indicated times; E: No microtubule aggregation in THP-1 and HUVEC during infection with *L*. *interrogans* strain Lai for the indicated times. F: Spot number of polymerized actin and tubulin. Bars show the means ± SD of three independent experiments. *: *p* < 0.05 *vs* the spot number of actin or tubulin which cells infected of 0h.

*L*. *interrogans* strain Lai could be found in cytosol of the THP-1 and HUVEC cells under transmission electron microscope after co-incubation of the spirochete with the host cells for 1 h (Fig 1A). The laser confocal microscopic results demonstrated a similar internalization of the spirochete into both the host cells in duration of infection for 1–4 h (Fig 1B and 1C). The data suggest that endocytosis is the major mechanism for internalization of *L*. *interrogans* into macrophages and vascular endothelial cells.

Rearrangement of microfilaments and microtubules is tested by host cells during internalization of *L*. *interrogans* strain Lai, the confocal microscopy and fluorescence of polymerized number results showed that only microfilaments in the THP-1 and HUVEC were significant aggregated during infection with *L*. *interrogans* strain Lai (Fig 1D, 1E and 1F).

### Various viability of intracellular *Leptospira* in THP-1 and HUVEC

**Fig 2:**
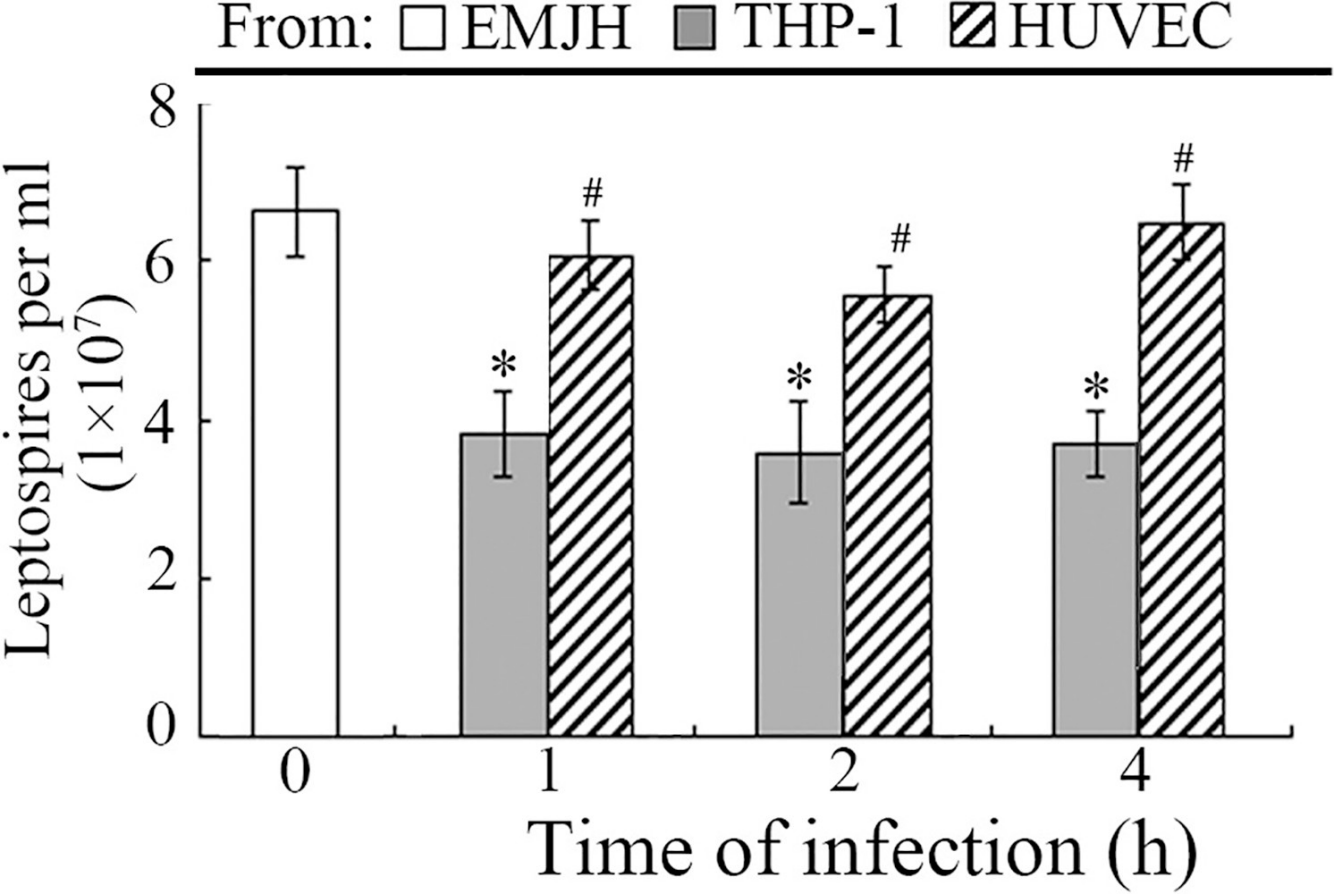
Various viability of *Leptospira* in THP-1 and HUVEC. Viability of *L*. *interrogans* strain Lai in the infected THP-1 and HUVEC for the indicated times. There is no significant difference between the numbers of leptospires in the cultures from specimens of the infected HUVEC and from EMJH medium. *: p < 0.05 vs the number of leptospires in the cultures from specimens of the EMJH medium. #: p < 0.05 vs the number of leptospires in the cultures from specimens of the infected THP-1 cells.

Briefly, internalized leptospires in both THP-1 and HUVEC cells were extracted, and same amount of leptospires from different cells were cultured in EMJH cultures for 6 days separately, the viability of leptospires were evaluated by calculating the number of leptospires in EMJH. The data suggest that the leptospires in HUVEC maintain high viability for proliferation in *vitro* than THP-1 (Fig 2) indicating that leptospires may be regulated by different cellular mechanisms in macrophages and vascular endothelial cells.

### Distribution of integrins and CAV-1 in THP-1 and HUVEC

**Fig 3:**
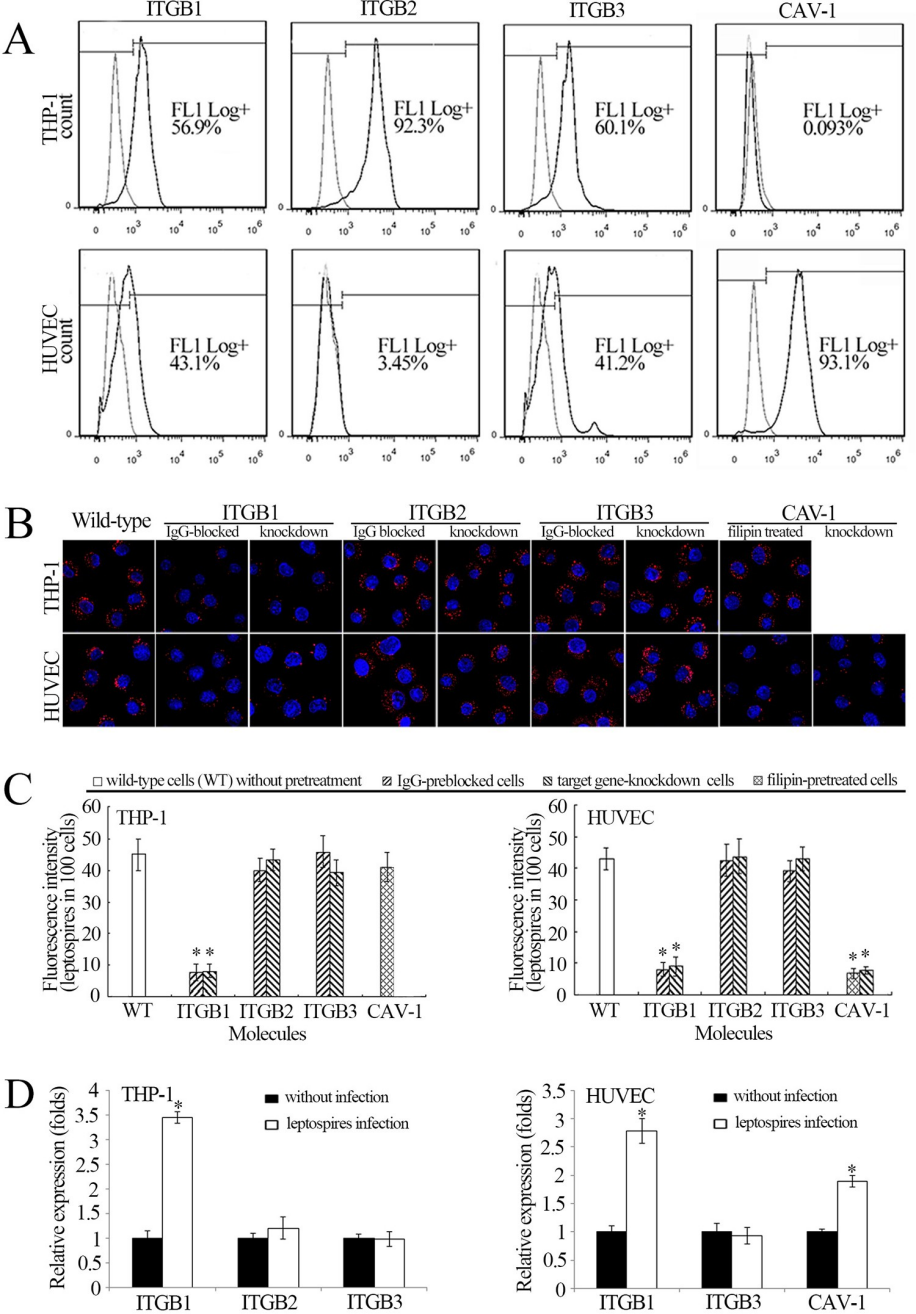
ITGB1 in THP-1 and CAV-1 in HUVEC responsible for internalization of leptospires. A: Distribution of ITGB1, ITGB2, ITGB3 and CAV-1 in THP-1 and HUVEC determined by flow cytometry. Ten thousand cells were analyzed for each of the specimens; B: Leptospires in THP-1 and HUVEC under the laser confocal microscope after infection with *L*. *interrogans* strain Lai for 1 h. The blue plaques in the middle of cells indicate the nuclus. The red spots around the nuclus indicate the intracellular leptospires; C: Statistical summary of red fluorescence intensity reflecting the leptospires in THP-1 and HUVEC after infection with *L*. *interrogans* strain Lai for 1 h. Statistical data from experiments such as shown in B. Bars show the means ± SD of three independent experiments. One hundred cells were analyzed for each of the specimens. *: *p* < 0.05 *vs* the red fluorescence intensity reflecting the leptospires in the wild type cells during infection. D: Relative gene expression of ITGB1, ITGB2 ITGB3, CAV-1 in THP-1 or HUVEC were analysed by comparing treatment of 4h of leptospires infection and non-infection. Bars show the means ± SD of three independent experiments. *: *p* < 0.05 *vs* the gene relative expression of treatment of non-infection.

The flow cytometric detection demonstrated that THP-1 expressed ITGB1, ITGB2 and ITGB3, but the CAV-1 was undetectable, However, the HUVEC was shown to express ITGB1, ITGB3 and CAV-1, but did not express ITGB2 (Fig 3A). The data suggest that there is a diversity of ITGB1, ITGB2, ITGB3 and CAV-1 distribution in macrophages and vascular endothelial cells.

### ITGB1 and CAV-1 responsible for internalization of *Leptospira* in THP-1 and HUVEC separately

Integrin subunit β1-, β2- or β3-encoding gene of THP-1 cells, integrin β1-, β2- or CAV-1-encoding gene of HUVEC were depleted using a siRNA Transfection Kit with the concentration of 100 nmol/L. The Real-time PCR (RT-qPCR) were carried out at 12h, 24h, 36h and 48h to confirmed the knockdown of target gene in THP-1 and HUVEC (S1 Fig). When the ITGB1 was blocked with ITGB1-IgG or the integrin β1 subunit-encoding gene were depleted with siRNA interference, the leptospires in the THP-1 during infection were significantly decreased compared to the wild-type cells (Fig 3B and 3C). In the HUVEC, the blockage of ITGB1 with antibody and inhibition of CAV-1 with filipin or depletion of ITGB1 and CAV-1 encoding genes with siRNA interference caused the noticeable decrease of intracellular leptospires during infection (Fig 3B and 3C). However, either the ITGB2 and ITGB3 blockage or intergrin β2 or β3 subunit-encoding gene knockdown did not affect the internalization of leptospires. The data suggest that the ITGB1 in THP-1 and both the ITGB1 and CAV-1 in HUVEC mediate the internalization of the spirochete during infection.

Relative gene expression of ITGB1, ITGB2 ITGB3 and CAV-1 in THP-1 or HUVEC were analysed by comparing treatment of 4h leptospires infection and non-infection. After infection of leptospires, the relative gene expression of ITGB1 in THP-1, ITGB1 and CAV-1 in HUVEC were up-regulated (Fig 3D).

### Phosphorylation of FAK or PI3K responsible for internalization of *Leptospira* in THP-1 and HUVEC separately

**Fig 4:**
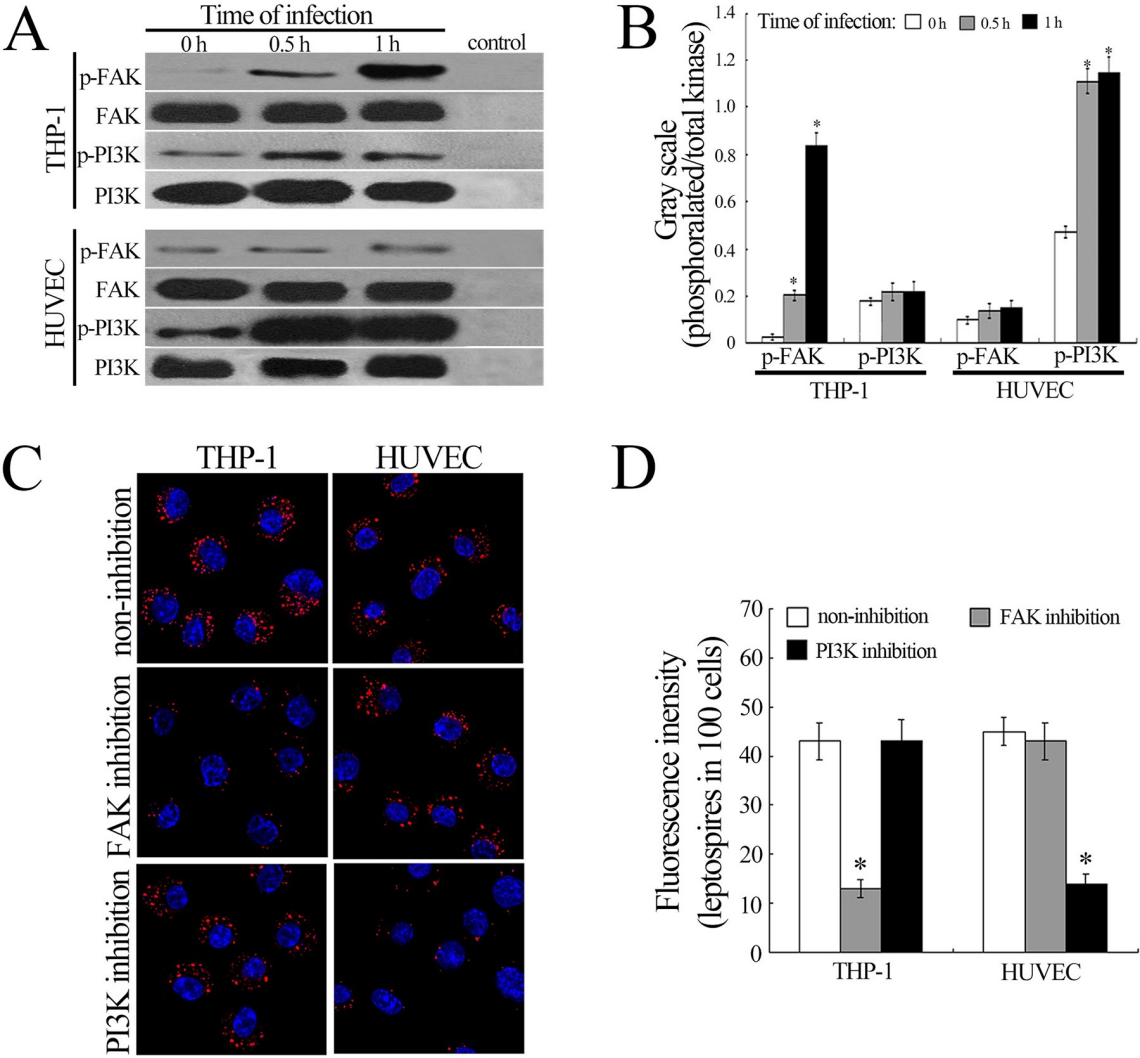
Phosphorylation of FAK and PI3K and cytoskeletal rearrangement in leptospire-infected cells. A: Phosphorylation of FAK in THP-1 and PI3K in HUVEC during infection with *L*. *interrogans* strain Lai for the indicated times; B: Quantification of immunoblotting bands reflecting FAK and PI3K phosphorylation in leptospire-infected THP-1 cells or HUVEC for the indicated times. Statistical data from experiments such as shown in A. Bars show the means ± SD of three independent experiments. *: *p* < 0.05 *vs* the phosphorylation level (gray scale) of FAK or PI3K in the cells before infection; C: Leptospires in FAK- or PI3K-inhibited THP-1 and HUVEC after infection with *L*. *interrogans* strain Lai for 1 h. The blue plaques in the middle of cells indicate the nuclus. The red spots around the nuclus indicate the intracellular leptospires; D: Statistical summary of red fluorescence intensity reflecting the leptospires in FAK or PI3K-inhibited leptospire-infected cells. Statistical data from experiments such as shown in C. Bars show the means ± SD of three independent experiments. One hundred cells were analyzed for each of the specimens. *: *p* < 0.05 *vs* the red fluorescence intensity reflecting the leptospires in the FAK-uninhibited THP-1 or PI3K-uninhibited HUVEC before infection.

FAK and PI3K signaling pathways have been shown to play crucial roles in cell membrane movement such as phagocytosis or internalization by regulation of cytoskeletal rearrangement [44]. However, our Western Blot assays confirmed that the phosphorylation of FAK, but not PI3K, in THP-1 occurred during infected with *L*. *interrogans* strain Lai (Fig 4A and 4B). In the leptospire-infected HUVEC, the PI3K rather than FAK was detectable for phosphorylation (Fig 4A and 4B). When the FAK in THP-1 were inhibited with FAK inhibitor-I or the PI3K in HUVEC was inhibited with PI3K inhibitor LY294002, the internalization of the spirochete was visibly attenuated (Fig 4C and 4D). However, the inhibition of FAK in HUVEC and PI3K in THP-1 had no influence on the leptospiral internalization (Fig 4C and 4D). The data indicate that there is a diversity of signaling pathways in macrophages and vascular endothelial cells to mediate the internalization of *L*. *interrogans*.

### Fusion of phagocytotic vesicles and lysosomes in THP-1 but not HUVEC

**Fig 5:**
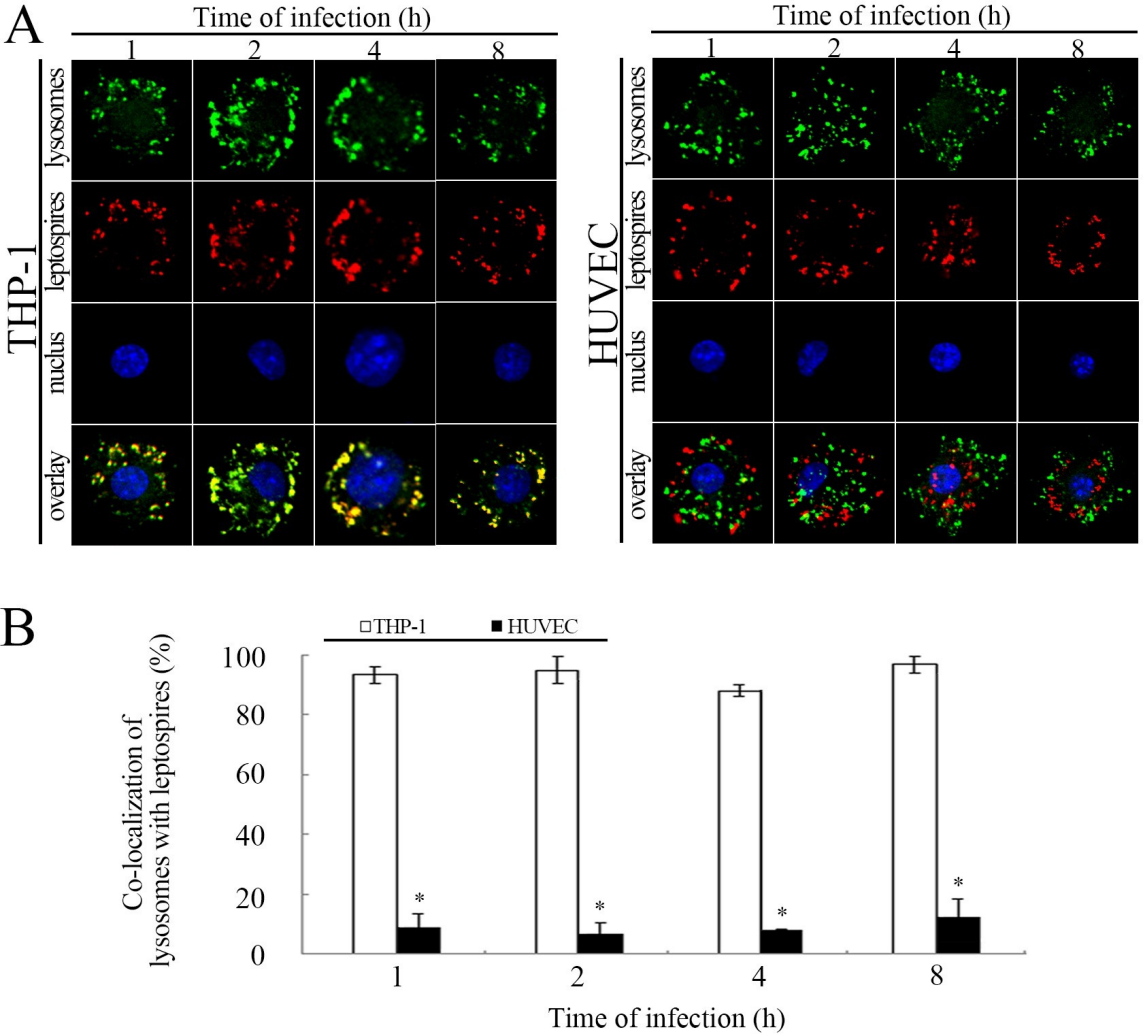
Fusion of intracellular leptospires with lysosomes or caveolae-based vesicles. A: Co-localization of phagocytotic vesicles with lysosomes (LAMP-1) in the THP-1 and HUVEC infected with *L*. *interrogans* strain Lai for the indicated times. The green spots marked with LAMP-1 indicate the lysosomes. The red spots indicate the intracellular leptospires. The yellow spots indicate the co-localization of the phagocytotic vesicles with the lysosomes; B: Co-localization percentages of phagocytotic vesicles with lysosomes in the THP-1 and HUVEC infected with *L*. *interrogans* strain Lai for the indicated times. Statistical data from experiments such as shown in A. Bars show the means ± SD of three independent experiments. One hundred cells were analyzed for each of the specimens. *: *P* <0.05 *vs* the co-localization percentages in the leptospire-infected THP-1 cells.

Phagocytes have been shown to kill the phagocytized bacteria by fusion of phagocytotic vesicles with lysosomes. Our confocal microscopic results showed that the leptospire-containing vesicles in THP-1 were co-localized with the lysosomes during infection with *L*. *interrogans* strain Lai (Fig 5A and 5B). Unexpectedly, no co-localization between the vesicles and lysosomes in the leptospire-infected HUVEC could be found (Fig 5A and 5B). The data suggest that the vascular endothelial cells, unlike macrophages, have a special mechanism to deal with the intracellular leptospires, and this mechanism facilitates the lossless transcytosis and transport of leptospires through blood vessels in the body.

### Powerful transcytosis ability of *Leptospira* through HUVEC monolayer using ITGB and caveolae

**Fig 6:**
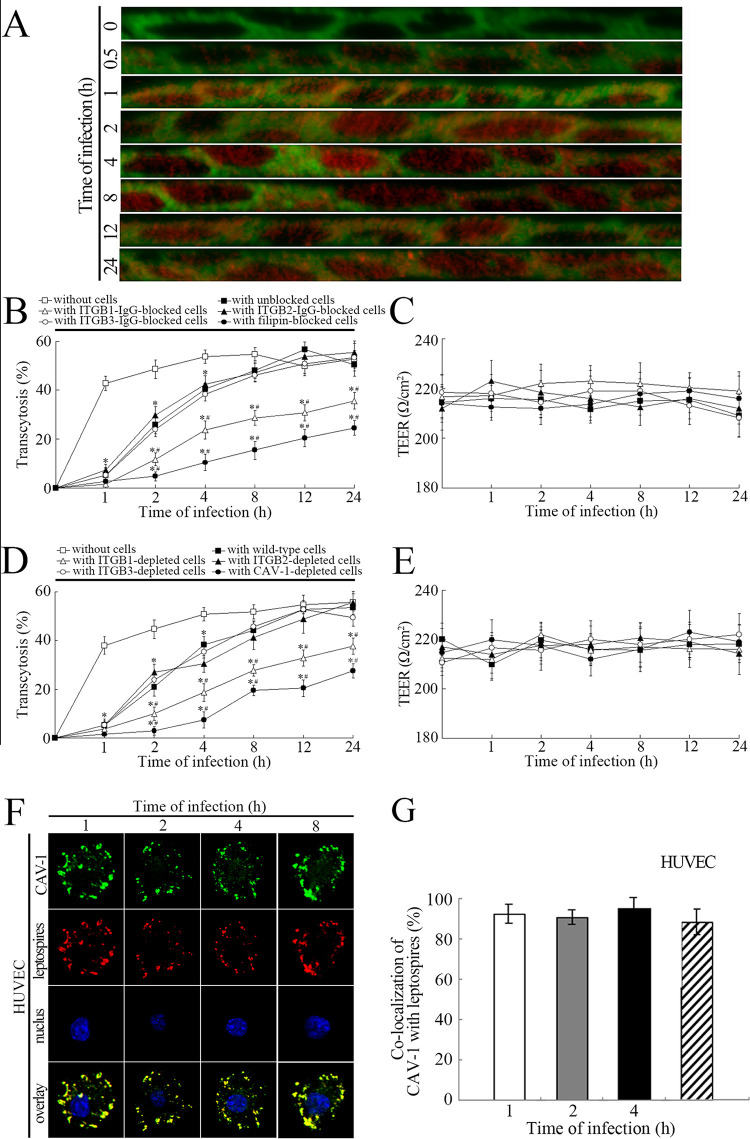
Powerful transcytosis ability of Leptospira through HUVEC monolayer. A: Laser confocal microscopic 3D-image reflecting *L*. *interrogans* strain Lai transmigrating HUVEC monolayer for the indicated times. The green plaques indicate the cell monolayers. The black plaques indicate the nuclus. The red spots indicate the intracellular leptospires; B: Transcytosis of *L*. *interrogans* strain Lai through HUVEC monolayer for the indicated times and transcytosis inhibition effects of ITGB1-IgG and filipin. *: *p* < 0.05 *vs* the transcytosis percentages of the spirochete transmigrating wells without cells. ^#^: *p* < 0.05 *vs* the transcytosis percentages of the spirochete transmigrating wild-type HUVEC monolayers; C: The TEER of HUVEC monolayer during transcytosis of *L*. *interrogans* strain Lai that shown in Fig 4B; D: Transcytosis of *L*. *interrogans* strain Lai through the intergrin β subunit or CAV-1 encoding gene depleted HUVEC monolayer for the indicated times. *: *p* < 0.05 *vs* the transcytosis percentages of the spirochete transmigrating wells without cells. ^#^: *p* < 0.05 *vs* the transcytosis percentages of the spirochete transmigrating wild-type HUVEC monolayers; E: The TEER of HUVEC monolayer during infection with *L*. *interrogans* strain Lai that shown in Fig 4D; F: Co-localization of intracellular leptospires with caveolae-based vesicles in the HUVEC infected with *L*. *interrogans* strain Lai for the indicated times. The green spots indicate the intracellular caveolae-based vesicles. The red spots indicate the intracellular leptospires. The yellow spots indicate the co-localization of the intracellular leptospires with the caveolae-based vesicles; G: Co-localization percentages of intracellular leptospires with caveolae-based vesicles in the HUVEC infected with *L*. *interrogans* strain Lai for the indicated times. Statistical data from experiments such as shown in F. Bars show the means ± SD of three independent experiments. One hundred cells were analyzed for each of the specimens.

The laser confocal microscopic 3D-image showed that *L*. *interrogans* strain Lai rapidly transmigrated into the HUVEC monolayer by invasion of cells or through cell junction (Fig 6A). The transwell test demonstrated that the spirochete could transmigrate through the HUVEC monolayer promptly with the maximal transcytosis percentages (52.7% and 56.6%) at the 12 h of post-infection (Fig 6B and 6D). In the whole infection process, the TEER of HUVEC monolayers were maintaining at the 208 to 203 Ω/cm^2^, indicating the integrity of monolayers (Fig 6C and 6E).

When the ITGB1 was blocked with antibody or the CAV-1 was inhibited with filipin, the transcytosis percentages of the spirochete was significantly decreased (Fig 6B). Moreover, the intergrin β1 subunit or CAV-1 encoding gene-depleted HUVEC also presented the much lower transcytosis percentages (Fig 6D). However, the ITGB2 and ITGB3 did not affect the transcytosis of leptospires (Fig 6B and 6D). The data suggest that powerful invasive *L*. *interrogans* transmigrate through vessel wall maintained high viability and the ITGB1 and CAV-1 mediate the transcytosis of the spirochete during infection.

The confocal microscopic indicated caveolae in cytosol of HUVEC could be co-localized with the intracellular leptospires during infection with *L*. *interrogans* strain Lai (Fig 6F and 6G). The data suggest that the caveolae in vascular endothelial cells mediates the internalization and transcytosis of *L*. *interrogans* which referring to the transport of the spirochete from blood stream into internal organs or tissues during infection. Caveolae has been confirmed to mediate the internalization of some certain bacteria into host cells and avoid fusion with lysosomes to protect pathogens from degradation [45].

## Discussion

Leptospirosis is a typical systemic infectious disease that relies on the powerful invasive ability of pathogenic *Leptospira* species [46]. After passing through human skin or a mucous membrane barrier, the spirochete invades the blood stream promptly. When in the blood, leptospires evade mononuclear-macrophages and resist phagocytosis [47]. Transmigration of the organism through small blood vessel walls occurs within several days to spread into nearly all of the internal organs to cause complicated clinical signs and symptoms [13]. Therefore, the resistance against phagocytosis of mononuclear-macrophages and transcytosis through vascular endothelial cells play crucial roles in successful systemic infection of leptospirosis.

Adherence is the prerequisite for bacterial pathogens to invade host cells. Fibronectin (FN), laminin (LN), collagen I (COL1) and COL4 are extracellular matrix (ECM) components that have been confirmed as the receptors for pathogenic *Leptospira* species [48,49]. The internalization of bacterial pathogens is mediated by a combination of ECM molecules with cell membrane integrins [26]. Caveolae (CAV), a cave-like structure in the cell membrane has also been shown to act as an entry-port for some certain bacteria [50]. Previous studies confirmed that among the CAV proteins (CAV-1, -2 and -3), CAV-1 plays a crucial role in structure and function of caveolae. Vascular endothelial cells, but not macrophages, express CAV-1 [51,52]. Our results demonstrate that THP-1 cells express all three integrin subfamily proteins (ITGB1, ITGB2 and ITGB3) except CAV-1, while HUVEC expressed ITGB1, ITGB3 and CAV-1 (Fig 3A). Interestingly, our Western Blot assays showed that the phagocytotic vesicles from the leptospire-infected THP-1 contained ITGB1 alone, while those from the infected HUVEC presented ITGB1 and CAV-1 (S2 Fig). In particular, only the inhibition of ITGB1 in the THP-1 or blockage of both the ITGB1 and CAV-1 in HUVEC could cause the significant decrease of intracellular leptospires during infection (Fig 3B and 3C). Interestingly, the relative expression of ITGB1 in THP-1 and ITGB1/CAV-1 in HUVEC was significantly up-regulated after infection of *L*. *interrogans* (Fig 3D). Moreover, the blockage of both the ITGB1 and CAV-1 resulted in the visible attenuation of leptospiral transcytosis through the HUVEC monolayers (Fig 6B and 6D). These data indicate that the internalization and transcytosis of *L*. *interrogans* is mediated by ITGB1 in macrophages or with both ITGB1 and CAV-1 in vascular endothelial cells.

The integrins αvβ3, αMβ2 and αLβ2 were also reported as pathogenic *Leptospira* receptors to activate intracellular signaling of cytoskeletal rearrangements, leading to microbial internalization [33,34]. Our study indicated the internalization of leptospires in THP-1 and HUVEC were F-actin dependent which is consistent with the previous results [53]. Previous studies showed macrophages contained *Pseudomonas aeruginosa* and Streptococcus pneumoniae infect macrophages by promotion of F-actin assembly. Pathogens such as *Listeria*, *Rickettsia*, *Burkholderia*, *Shigella* and *Mycobacteria* subvert cellular actin dynamics to facilitate their movement within the host cytosol and promote their intracellular survival [54, 55]. During bacterial internalization, the formation of integrin dimerization caused by combination with ECM molecules has been shown to trigger FAK and PI3K signaling pathways [56]. The integrin aggregation can also stimulate CAV-1 phosphorylation which induces the activation of the PI3K signaling pathway [57]. Both FAK and PI3K pathways have been confirmed to mediate the phagocytosis or internalization of bacteria by microfilament- and/or microtubule-dependent cytoskeletal rearrangement [58]. However, in the leptospire-infected THP-1 cells, only FAK activation and microfilament aggregation were found, while the leptospire-infected HUVEC resulted in activation of PI3K and rearrangement of microfilament (Figs 4A and 1D). Importantly, inhibition of either FAK in the macrophages or PI3K in the HUVEC resulted in a noticeable attenuation of leptospiral internalization (Fig 4C and 4D). These data indicate that the FAK or PI3K pathway is responsible for microfilament rearrangement signaling in macrophages or vascular endothelial cells during the internalization of *L*. *interrogans*. Both PI3K and FAK can mediate cytoskeleton rearrangement for pathogen internalization into host cells, but PI3K mediated the invasion of *Escherichia coli* into vascular endothelial cells, while FAK mediated the entry of *Salmonella typhimurium* into fibroblasts [59,60].

The diverse mechanisms of mediating the endocytosis of *L*. *interrogans* may be due to the different types of host cells. In the present study, we found that the leptospires in HUVEC co-localized with intracellular caveolae but not with lysosomes (Figs 5A and 6F), intracellular leptospires from the HUVEC had more powerful reproductive ability in medium than that from the THP-1 cells (Fig 2). Furthermore, the leptospires passed through the HUVEC monolayers and presented typical morphology and motility under the dark-field microscope. This phenomenon has been described in several previous reports, and the possible mechanism may be related to a bacterial defense mechanism when inside caveolae that avoids fusion with lysosomes to protect pathogens from degradation [45]. In addition, we found that ITGB1 and CAV-1 also play important roles in transcytosis of *L*. *interrogans*. Upon inhibition and gene-depletion of ITGB1/CAV-1, the transcytosis efficiency of *L*. *interrogans was* reduced (Fig 6B and 6D). In human enteric, renal, and umbilical vein endothelial cell lines, *L*. *interrogans* was shown to use M16-Type metallopeptidases to hydrolyze extracellular matrix proteins and disassemble cell apical junctional complexes (AJCs), which may enhance transmigration through cell monolayers [61, 62].

**Fig 7:**
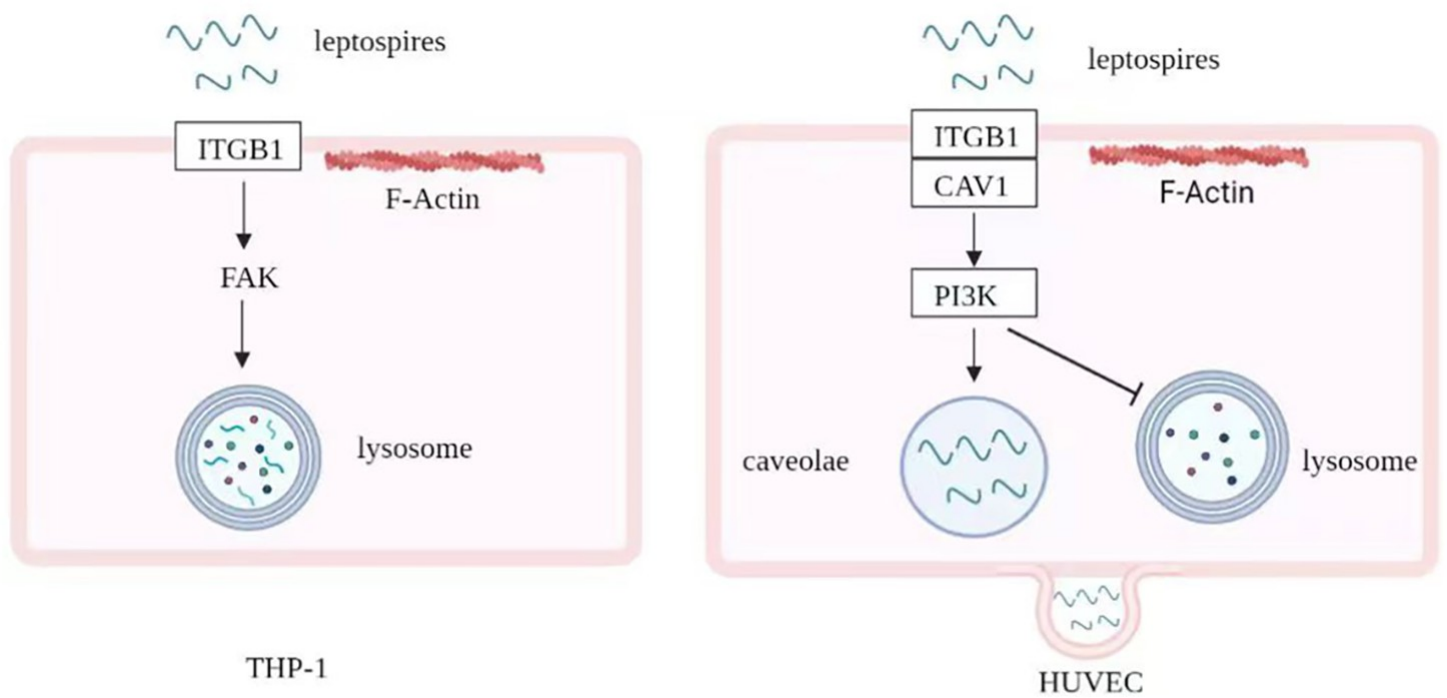
Graphical abstract of diverse internalization mechanisms of *Leptospira interrogans* in human macrophages and vascular endothelial cells.

Taken together, our findings indicate that ITGB1/FAK-signaling and ITGB1/CAV-1/PI3K-signaling mediate the F-actin dependent internalization and transcytosis of *L*. *interrogans* in macrophages and vascular endothelial cells, respectively, which affect the outcomes for both the pathogen and host cells (Fig 7).

## Supporting information

S1 Fig Detection of relative expression of target gene in THP-1 and HUVEC by real-time PCR. Integrin subunit β1-, β2- or β3-encoding gene of THP-1 cells, integrin β1-, β2- or CAV-1-encoding gene of HUVEC were depleted using siRNA Transfection Kit. The Real-time PCR (RT-qPCR) were carried out at 12h, 24h, 36h and 48h to confirmed the knockdown of target gene in THP-1 and HUVEC, the results indicated target genes were significant knocked down by siRNA treatment.
(TIF)

S2 Fig Distribution in ITGB1 and CAV-1 in the phagocytotic vesicles of THP-1 and HUVEC during infection with L. interrogans strain Lai determined by Western Blot assay. ITGB1, ITGB2, ITGB3 and CAV-1 in the phagocytotic vesicles of THP-1 and HUVEC were detected during infection with L. interrogans strain Lai, phagocytotic vesicles from the leptospire-infected THP-1 contained ITGB1 alone, while those from the infected HUVEC presented ITGB1 and CAV-1.
(TIF)
