# Supplementary figures and images for: Internalization of Leptospira interrogans via diverse endocytosis mechanisms in human macrophages and vascular endothelial cells
Source: PLoS Negl Trop Dis. 2022 Sep 22;16(9):e0010778. doi: 10.1371/journal.pntd.0010778 (PMC9531806; doi:10.1371/journal.pntd.0010778)

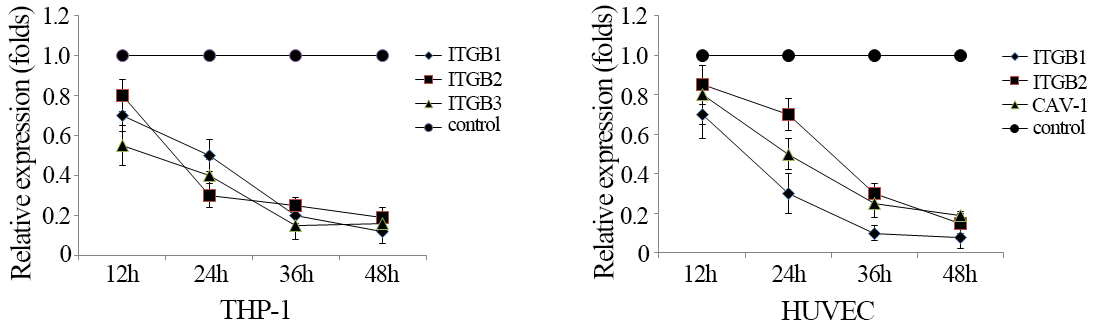

Supplement: S1 Fig — Integrin subunit β1-, β2- or β3-encoding gene of THP-1 cells, integrin β1-, β2- or CAV-1-encoding gene of HUVEC were depleted using siRNA Transfection Kit. The Real-time PCR (RT-qPCR) were carried out at 12h, 24h, 36h and 48h to confirmed the knockdown of target gene in THP-1 and HUVEC, the results indicated target genes were significant knocked down by siRNA treatment. (TIF) [file pntd.0010778.s001.tif]

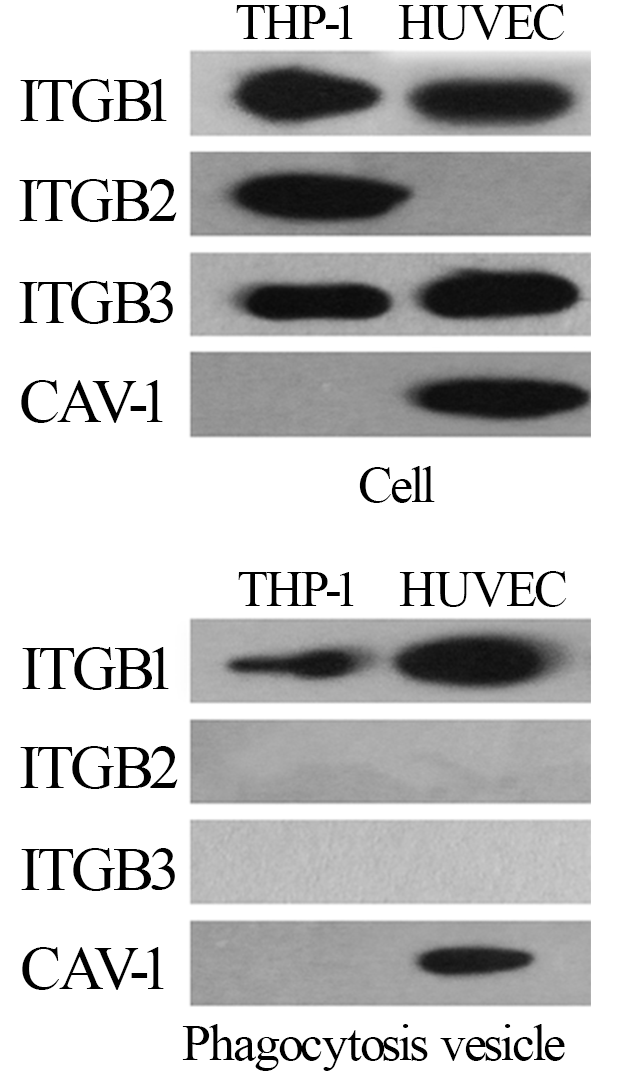

Supplement: S2 Fig — ITGB1, ITGB2, ITGB3 and CAV-1 in the phagocytotic vesicles of THP-1 and HUVEC were detected during infection with L. interrogans strain Lai, phagocytotic vesicles from the leptospire-infected THP-1 contained ITGB1 alone, while those from the infected HUVEC presented ITGB1 and CAV-1. (TIF) [file pntd.0010778.s002.tif]
